# Increasing Antibody Responses to Five Doses of SARS-CoV-2 mRNA Vaccine in Lung Transplant Patients

**DOI:** 10.3390/jcm12124125

**Published:** 2023-06-18

**Authors:** Johanna van Gemert, Fleur Steenberg, Coretta van Leer-Buter, Huib Kerstjens, Willie Steenhuis, Onno Akkerman, Erik Verschuuren, Tji Gan

**Affiliations:** 1 Department of Pulmonary Diseases, Tuberculosis and Lung Transplantation, University of Groningen, University Medical Center Groningen, 9713 GZ Groningen, The Netherlands; f.j.steenberg@umcg.nl (F.S.); h.a.m.kerstjens@umcg.nl (H.K.); w.n.steenhuis@umcg.nl (W.S.); o.w.akkerman@umcg.nl (O.A.); e.a.m.verschuuren@umcg.nl (E.V.); c.t.gan@umcg.nl (T.G.); 2 Department of Virology, University of Groningen, University Medical Center Groningen, 9713 GZ Groningen, The Netherlands; c.van.leer@umcg.nl

**Keywords:** COVID-19, vaccine, lung transplantation

## Abstract

Purpose: COVID-19 causes high mortality in Lung Transplant (LTx) patients, therefore vaccination in this population is potentially life-saving. However, the antibody response is impaired after three vaccinations in LTx patients. We questioned whether this response might be increased, and therefore studied the serological IgG antibody response across up to five doses of the SARS-CoV-2 vaccine. In addition, risk factors for non-response were investigated. Methods: In this large retrospective cohort study, antibody responses were assessed after 1–5 mRNA-based SARS-CoV-2 vaccines in all LTx patients between February 2021 and September 2022. A positive vaccine response was defined as an IgG level ≥ 300 BAU/mL. Positive antibody responses due to COVID-19 infection were excluded from the analysis. Outcome and clinical parameters were compared between responders and non-responders, and multivariable logistic regression analysis was performed to determine the risk factors for vaccine-response failure. Results: The antibody responses of 292 LTx patients were analyzed. Positive antibody response to 1–5 SARS-CoV-2 vaccinations occurred in 0%, 15%, 36%, 46%, and 51%, respectively. During the study period, 146/292 (50%) of the vaccinated individuals tested positive for SARS-CoV-2 infection. The COVID-19-related mortality was 2.7% (4/146), and all four patients were non-responders. Risk factors associated with non-response to SARS-CoV-2 vaccines in univariable analyses were age (*p* = 0.004), chronic kidney disease (CKD) (*p* = 0.006), and shorter time since transplantation (*p* = 0.047). In the multivariable analysis, they were CKD (*p* = 0.043), and shorter time since transplantation (*p* = 0.028). Conclusion: A two- to five-dose regime of SARS-CoV-2 vaccines in LTx patients increases the probability of vaccine response and results in a cumulative vaccine response in 51% of the LTx population. LTx patient antibody response to SARS-CoV-2 vaccinations is therefore impaired, especially in patients shortly after LTx, patients with CKD, and the elderly.

## 1. Introduction

Since the beginning of the COVID-19 pandemic, highly effective SARS-CoV-2 vaccines have been developed [1,2]. The rapid development and distribution of vaccines has proved to be instrumental in reducing the incidence and severity of COVID-19 in the general population [1,3,4]. However, most of the initial trials excluded solid organ transplant (SOT) patients [1,3,5]. Unfortunately, SOT patients are less likely to develop an antibody response to SARS-CoV-2 vaccines and have a higher risk of worse COVID-19 disease course and outcome, due to their immunosuppressive status and additional comorbidities [6,7,8]. SOT patients elicit a reduced immunogenicity to COVID-19 vaccines, because of the inhibition of lymphocyte activation, interaction with antigen-presenting cells, and decreased B-cell memory responses, depending on the type of immunosuppressive drug [6]. Therefore, these patients must reach an optimal vaccination status to protect them against severe COVID-19. Antibody response to SARS-CoV-2 vaccines was reported to vary according to the type of SOT, and the response was especially low in lung transplant (LTx) patients [9]. Antibody response in LTx patients is likely to be lower than in other SOT patients because LTx patients receive more intensive immunosuppression than other SOT patients. Several studies have investigated the development of an immune response after receiving two or three vaccines in LTx patients and have shown poor antibody response even after a third vaccine (16–46%) [10,11]. We hypothesize that more LTx patients might achieve antibody response after a fourth or fifth vaccine. Therefore, the primary objective of this study is to document increasing humoral antibody responses after one to five vaccine doses. The secondary objective is to identify the patient characteristics that predict both reduced and increased humoral responses to SAR-COV-2 vaccination in LTx patients. Additionally, the study aims to investigate any potential differences in disease course between vaccine responders and non-responders.

## 2. Material and Methods

### 2.1. Patients

Data from LTx patients were retrospectively collected at the University Medical Center Groningen, the Netherlands, between February 2021 and September 2022. All SARS-CoV-2 vaccinated adult LTx patients, with at least one measured antibody response between February 2021 and September 2022, were eligible for inclusion. Patients were excluded if they had received one or more vaccinations before the transplantation. Patients were also excluded if the number of vaccines given or the date of the vaccination or antibody measurement was unknown. Only mRNA-based SARS-CoV-2 vaccines were used in all patients, i.e., Moderna or Pfizer/BioNTech (Comirnaty) according to the COVID-19 treatment guidelines, special considerations in SOT patients. Furthermore, in accordance with these guidelines, the COVID-19 vaccines were offered as early as 3 months after transplantation, and after receiving the primary series or booster doses. LTx patients were advised to continue to take precautions [12]. For classifying chronic kidney disease (CKD), we used the Kidney Disease Improving Global Outcomes (KDIGO) staging [13]. Heart failure was classified according to the 2021 ESC guidelines [14]. 

### 2.2. Transplant Care

Follow-up of all patients took place at least every 3 months for the monitoring of transplant function, with spirometry and blood tests. Patients received standard maintenance immunosuppression: tacrolimus, prednisolone, and mycophenolate mofetil (MMF), and induction with Basiliximab postoperatively. Alternative immunosuppressants were cyclosporine, azathioprine, everolimus, or sirolimus. Tacrolimus trough level goals were specified per protocol at 7–10 ng/mL. Post-transplantation prophylactic therapy included cotrimoxazole for pneumocystis pneumonia and valganciclovir/aciclovir for CMV, depending on CMV (mis)match. The management of COVID-19 with steroids, adjustment of immunosuppression, and COVID-19-directed therapies was according to the ISHLT Guidance [15]. Patients were urged to contact the transplant team on a low-threshold basis when infected with SARS-CoV-2.

### 2.3. S-Specific Antibody Testing

Humoral responses to vaccination were measured with a quantitative IgG assay detecting antibodies against the SARS-CoV-2 Spike (s) protein, with a lower limit of detection of 50 AU/mL. Antibody responses to vaccination were measured after standard outpatient visits, at least every 3 months. The same IgG test was used for all participants, i.e., the SARS-spike-IgG quantitative test by Abbott, on the Aliniti-I platform. The assay was performed following the manufacturer’s instructions [16]. A positive vaccine response was defined as an IgG level above 300 AU/mL, and a low response as an IgG level between 50 and 300 AU/mL [17,18]. Antibody responses were excluded from the analysis if they developed after a positive SARS-CoV-2 test, as these antibody responses could be due to COVID-19 and therefore could not be considered (solely) a vaccine response. Intercurrent infections were diagnosed by PCR. If PCR tests were repeatedly negative and there were clinical suspicions of a recent COVID-19 infection, antibody testing of Nucleocapsid (N) antibodies was performed using the SARS-N antibody qualitative IgG test on the Abbott Aliniti-I. platform.

### 2.4. Patient Groups

Responders were defined as patients with a positive SARS-CoV-2 antibody (≥300 BAU/mL); non-responders were defined as patients without a SARS-CoV-2 antibody response (<50 BAU/mL); low responders were defined as patients with a SARS-CoV-2 antibody response between 50 and 300 BAU/mL. Patients with a previously negative vaccine antibody response, but a positive antibody response after a positive SARS-CoV-2 antigen test were defined as “post-COVID-19 responders” and were excluded from analysis.

Patients who tested positive for SARS-CoV-2 antibodies after a given vaccine were considered to be responders to a subsequent vaccine. Patients who were non-responders after a given vaccine were considered unknown if their antibodies were not measured after a subsequent vaccine.

### 2.5. Statistical Analysis

Analyses were carried out using IBM SPSS for Mac, version 24.0. Continuous variables are expressed as median (interquartile range). After each vaccination, the number of patients with a positive response was measured cumulatively. Wilcoxon Rank tests were used for within-group analyses to compare the number of patients with and without IgG response after 1–5 vaccines. Clinical parameters and outcomes were compared between responders and non-responders by univariable analysis (Mann–Whitney U-test and chi-squared test). All parameters with a *p*-value less than 0.10 by univariable analysis were considered to be a trend to statistical significance and were entered into a multivariable logistic regression analysis model, to identify independent risk factors for vaccine-response failure. A *p*-value of less than 0.05 was considered significant. Risk factors are expressed as odds ratios with 95% confidence intervals and *p*-values.

## 3. Results

### 3.1. All Patients

Of the 832 patients transplanted between November 1990 and September 2022, 406 patients were alive at the beginning of the study period. Of those, 107 patients were excluded for the following reasons: vaccinated before LTx (53/107; 50%), no vaccination (23/107; 21%), number of vaccines/date of the vaccination unknown 17/107 (16%), children 9/107 (8%), no antibody measurement (3/107; 3%), loss to follow-up (2/107; 2%). A total of 292 (72%) of the alive adult LTx patients were included in the study. Baseline characteristics for all 292 LTx patients with antibody responses after 2–5 vaccinations are shown in Table 1. The median age was 60 years (IQR 48–66) and most patients (52%) were male. COPD (41%) and pulmonary fibrosis (21%) were the most common indications for LTx, and a bilateral LTx was performed in 91%. The median time between LTx and the first vaccine was 8 years (IQR 4–12). The most frequently reported comorbidities were dyslipidemia (81%), CKD (70%), and hypertension (47%). During the study, 20 (7%) of the LTx patients died, 4 (1%) from COVID. Details of the responders and non-responders are shown in Table 1. In general, responders were younger, had a longer interval since transplantation, and less often had CKD. In Supplement 1, clinical parameters of the excluded post-COVID-19 responders are included. 

### 3.2. Responders

In the responders group, 56% of the 132 patients were male, the median age was 58 years (IQR 40–64), and 96% were transplanted bilateral (Table 1). The most common indications for LTx in the responders group were COPD (42%); fibrosis (19%), and CF (19%). The median time between LTx and the first vaccine was 9 years (IQR 5–13). The most frequently reported comorbidities were dyslipidemia (82%), CKD (63%), and hypertension (44%). During the study period, 3 (2%) of the responders died, none from COVID-19.

### 3.3. Non-Responders

In the non-responders group, 51% of the 126 patients were male, the median age was 62 years (54–66), and 90% were transplanted bilateral (Table 1). The most common indications for LTx in the non-responders group were COPD (42%) and fibrosis (25%). The median time between LTx and the first vaccine was 7 years (IQR 4–11). The most frequently reported comorbidities were dyslipidemia (79%), CKD (79%), and hypertension (51%). During the study period, 17 (14%) of the non-responders died, and 4 (3%) from COVID-19.

### 3.4. Vaccines

In 292 SARS-CoV-2-vaccinated LTx patients, 100% received 1 vaccine, 98% received 2 vaccines, 90% received 3 vaccines, 72% received 4 vaccines, and 46% received 5 vaccines. Five patients died before the second vaccination. The median time between the first and second vaccine was 1 month (IQR 0–1), between the second and third vaccine 6 months (IQR 5–6); between the third and fourth vaccine 2 months (2–3) and between the fourth and fifth vaccine 3 months (IQR 2–3). The cumulative positive antibody response rate to SARS-CoV-2 vaccination was 0% in patients with measured antibody response after 1 vaccination, 15% (37/249) after 2 vaccinations, 36% (85/234) after 3 vaccinations, 46% (112/243) after 4 vaccinations and 51% (113/220) after 5 vaccinations (Table 2). The median time between vaccination and antibody measurement was 16 weeks (IQR 3–20) after the first vaccine, 15 weeks (IQR 11–18) after the second vaccine, 5 weeks (IQR 4–9) after the third vaccine, 5 weeks (IQR 4–9) after the fourth vaccine and 8 weeks (IQR 4–13) weeks after the fifth vaccine. The median number of vaccines needed before a positive antibody response (in those with a response) was 3 (IQR 2–4).

**Figure 1:**
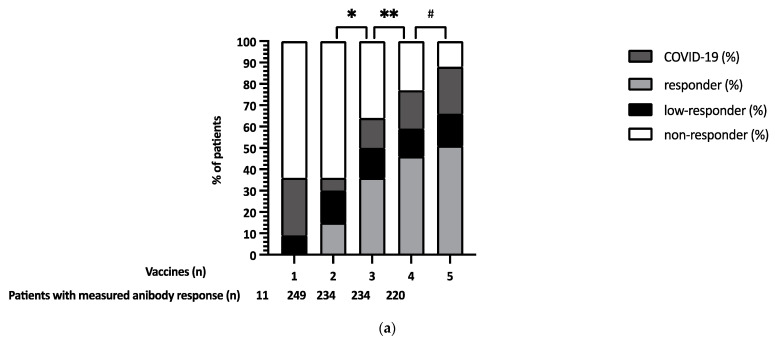

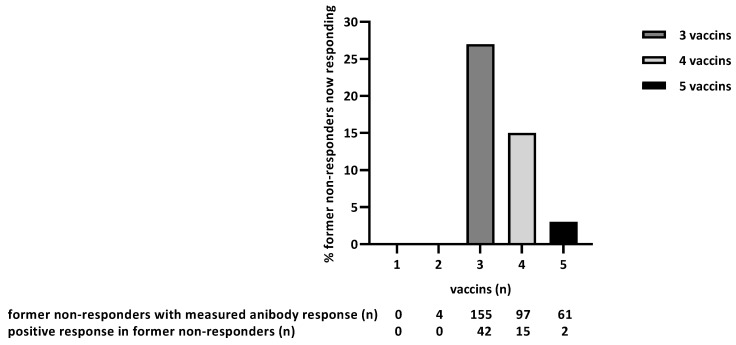
(**a**) SARS-CoV-2-specific cumulative antibody response after 1–5 vaccinations in LTx patients. * *p*-value < 0.001 for the difference between responders after the 2nd and 3rd vaccination. ** *p*-value < 0.001 for the difference between responders after the 3rd and 4th vaccination. ^#^ *p*-value 0.21 for the difference between responders after the 4th and 5th vaccination. (**b**) Percentage of former non-responders responding after 1–5 SARS-CoV-2 vaccines in LTx patients.

**Figure 2:**
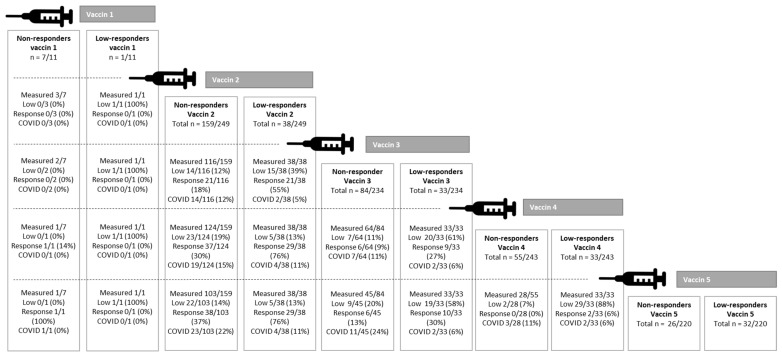
Non-responders and low responders after 1–5 SARS-CoV-2 vaccinations in LTx patients.

The number of patients with a positive antibody response increased after each vaccination, starting from vaccination 2 (Figure 1a,b). A total of 42/154 (27%) of the non-responders after the second vaccine became responders after the third vaccine; 15/97 (15%) of the non-responders after the third vaccine became responders after the fourth vaccine; 2/61 (3%) of the non-responders after the fourth vaccine became responders after fifth vaccine (Figure 1b). Figure 2 shows the responses of the non-responders and low responders after 1–5 vaccines. With the help of Figure 2, an attempt can be made to estimate the likelihood that a non-responder will become a responder after further vaccination. For example, a considerable percentage of low responders after 2 vaccines became responders after the third or fourth vaccine (55% and 76%). Similarly, 18 and 30% of non-responders after 2 vaccines became responders after a third or fourth vaccine. The addition of a fifth vaccine in non-responders or low responders after a fourth vaccine was only useful for a small number (3%) of LTx patients (Figure 1b and Figure 2). After 5 vaccines, 51% of the total population had an antibody response.

### 3.5. Risk Factors for the Failure to Develop an Antibody Response after Vaccination

Patient characteristics were compared between vaccine responders and non-responders (Table 1). Risk factors associated with non-response to SARS-CoV-2 vaccines were CKD (*p* = 0.006), older age (*p* = 0.004), and shorter time between LTx and the initial vaccine (*p* = 0.047) in univariable analysis. In a multivariable analysis, CKD (*p* = 0.043; OR = 1.9; 95% CI [1.02–3.57]) and a shorter time between LTx and the initial vaccine (*p* = 0.028; OR = 1.1; 95% CI [1.00–1.10]) were independently associated with vaccine-response failure.

### 3.6. COVID

During the study period, 146/292 (50%) of the vaccinated LTx patients tested positive for SARS-CoV-2 (Table 1). When infected with COVID-19, 50% of the patients received an increased dosage of steroids because of severe symptoms, the need for supplemental oxygen, or a decline in home spirometry. Antimetabolites were discontinued or reduced by 48%. Tacrolimus trough levels were maintained in all patients. Of the 123 responders, 49 (40%) patients developed COVID-19 during the study period, while 54 (43%) of the non-responders developed COVID-19 (*p* = 0.629). Of the responders, 4% tested positive for SARS-CoV-2 after the first vaccine, 10% after the second vaccine, 33% after the third vaccine, 43% after the fourth vaccine, and 10% after the fifth vaccine. Of non-responders, 2% tested positive for SARS-CoV-2 after the first vaccine, 15% after the second vaccine, 15% after the third vaccine, 37% after the fourth vaccine, and 31% after the fifth vaccine. The mortality rate associated with COVID-19 was 2.7% (4/146). All 4 patients who died were non-responders. Of the 146 patients with COVID, 20 (14%) were admitted. The criteria for hospitalization were clinical symptoms of (upper) respiratory infection, fever, or shortness of breath with or without oxygen requirement (peripheral oxygen saturation < 92%) [19]. There was no significant difference in COVID-19-related admission rates between responders and non-responders (Table 3). 

### 3.7. All-Cause Mortality

Twenty patients died during the study period. The causes of death were cancer (7/20; 35%), CLAD (4/20; 20%), COVID-19 (4/20; 20%), renal failure (3/20; 15%), subarachnoid hemorrhage (1/20; 5%), and lung bleeding 1/20; 5%). In the 20 LTx patients who died during the study period, 35% had received 1 vaccine, 50% had received 2 vaccines, 10% had received 3 vaccines, 5% had received 4 vaccines, and none had received 5 vaccines. The all-cause mortality was significantly higher in non-responders (14%), compared to responders (2%) (*p* = 0.001). 

## 4. Discussion

To our knowledge, this is the first study in a large LTx population that describes the antibody response after receiving 1–5 SARS-CoV-2 vaccines. We showed that the number of patients with a positive antibody response increases with the number of vaccines received and increases by up to 51% after five vaccines. Patients without an antibody response or with low antibodies after a second vaccination have a significant chance of developing antibodies after a third or fourth vaccination and a 3% chance after a fifth vaccine. This is consistent with the case series of Alejo et al., who showed in a small study of 18 patients with any SOT and without an antibody response after a third vaccine, 50% developed a response after the fourth vaccine. All patients with a low response after the third vaccination had a good response after the fourth vaccination [20]. In addition, Abedon et al. showed that 18 SOT patients with a low antibody response after a fourth vaccination had more antibodies after a fifth vaccination [21]. The vaccination response in LTx patients after two vaccines remains lower than seen in liver-, heart-, and kidney-transplant patients, most likely due to the higher levels of immunosuppression used in LTx patients compared to other SOT patients [8,18]. Our large study in LTx patients, in addition to the smaller studies in SOT patients, advocates the importance of measuring an antibody response in LTx patients following SARS-CoV-2 vaccination, since there is a significant chance of developing antibodies after a third or fourth vaccine, and even some chance after a fifth vaccine in the case of a prior negative antibody response. However, the increase in response rate is smaller with each additional vaccine after the third, suggesting that a significant number of LTx patients may not develop antibodies, even when multiple doses are given. 

Repeated vaccinations with SARS-CoV-2 mRNA vaccines have been shown to be safe, not causing major adverse effects in SOT patients [1,10].

We have shown that in LTx, patient age, CKD, and a shorter time since transplantation were associated with the failure to obtain an antibody response after SARS-CoV-2 vaccination in univariable analysis. In multivariable analysis, CKD and a shorter time since transplantation were independently associated with vaccine-response failure. Patients without a response after vaccination were older, more often had CKD, and had a shorter period of time after LTx. Age-related heterogeneity of SARS-CoV-2 vaccine-acquired immunity is not specific to LTx patients, but has also been seen in the general population [22]. Studies in SOT patients have also found that older age is associated with a failure to obtain an antibody response in SOT patients [8,10,23,24]. CKD is also a known risk factor for vaccine failure in non-SOT and SOT patients. In non-SOT CKD patients, the efficacy of vaccines might be hampered because of the alteration of immunity by the uremic milieu, vitamin D, and EPO deficiency [22]. In studies with SOT patients, CKD has been associated with a reduced immune response to SARS-CoV-2 vaccination [25,26]. 

We showed that a shorter time between LTx and the first vaccine was associated with antibody response failure after SARS-CoV-2 vaccination. In the first year after LTx, immunosuppression is most intense. Patients who have had LTx more recently will therefore have a more intense immunosuppressive regimen and probably a lower chance of a vaccine response. This might explain why a shorter time after LTx is associated with vaccine-response failure. Such an association has also been shown by Hoek et al. They showed in their study that a lower torque tenovirus (TTV) load, which is associated with less-intensive immunosuppression and a longer time since transplantation, is associated with a better antibody response to SARS-CoV-2 vaccines [18]. In our study, there was no difference in the type of immunosuppressive drug’s impact on antibody response. Other studies have shown that only MMF is significantly associated with vaccine failure. MMF is a potent inhibitor of B-cell function and immunoglobulin secretion [10,24,27]. However, we could not confirm this association in our study. This could be because most people (76%) in our study used MMF.

Although repeated SARS-CoV-2 vaccination in LTx patients results in a better antibody response, this does not prevent SARS-CoV-2 infection. The percentage of COVID-19 cases was not higher in non-responders than in responders (43% vs. 40%, *p* = 0.629). The lack of a significant difference may be due to the possibility that non-responders may protect themselves from infection better than responders using masks, good hand hygiene, social distancing, etc. Another explanation could be due to the timeframe of this study, during which various COVID variants emerged, against which the available vaccines were not equally effective. In our earlier COVID-19 study in LTx patients, when vaccines were not available and the Delta variant was endemic, the hospitalization rate was 57% and the mortality rate was 20% [19]. In our current study, admission rates and mortality due to COVID-19 are much lower (14% and 4%). This is not only due to the availability of SARS-CoV-2 vaccines, but might also be due to the less-virulent Omicron variant and more knowledge about treating COVID-19 in immunocompromised patients, such as administering monoclonal antibodies in patients without antibodies. Admission rates were not significantly higher in non-responders than in responders. However, all four patients who died from COVID-19 in this study were non-responders.

The all-cause mortality was significantly higher in non-responders, compared to responders. However, the vaccination rate among deceased patients was low because they died before their next vaccination during the study period. Therefore, deceased patients are less likely to have been vaccine responders.

This study has some limitations. Because it is a retrospective study, we did not have complete antibody assessments in all patients after each vaccination. Nevertheless, our study does provide a reliable reflection of daily practice regarding vaccination in immunocompromised patients. Additionally, we did not measure T-cell responses, which may be detectable even in patients without antibody response. Accumulating evidence has shown the importance of T-lymphocyte responses in protection against severe COVID-19 [28,29,30]. Our finding that serological non-responders did not require hospital admission more frequently than serological responders may, to some extent, be attributable to a degree of vaccination-induced T-cell immunity which we did not measure. Lastly, we did not examine the effect of different mRNA vaccines on obtaining an antibody response.

Our data show that, in LTx patients, it is important to vaccinate repeatedly with the probability of a vaccine response increasing but reaching a plateau after about 4–5 vaccinations. In the group of LTx patients with no response after 4–5 vaccinations, who are older, have renal insufficiency, and are shorter after transplantation, the likelihood of developing a vaccine response after further vaccination is low. In these patients, the adjustment of immunosuppression to lower doses and/or lower trough levels could be considered to increase the likelihood of a vaccine response. However, further studies are needed to confirm this.

## 5. Conclusions

A two- to five-dose regime of SARS-CoV-2 vaccines in LTx patients increases the probability of vaccine response, reaching a plateau after about four to five vaccinations. There is still a group of patients who are older, have CKD, and are more recently post-transplant who are unlikely to respond even after five vaccinations. Therefore, repeated vaccination and measurement of antibody responses after each SARS-CoV-2 vaccination is important, particularly in those at risk of vaccine failure. 

## Figures and Tables

**Table 1:** Baseline characteristics of all SARS-CoV-2 vaccinated LTx patients, responders and non-responders.

Variable *n*, (%)	All Patients ^#^ 292	Responders 123 (42)	Non-Responders 126 (43)	*p*-Value *
Age, years	60 (48–66)	58 (40–64)	62 (54–66)	0.004
Gender, male (%)	152 (52)	69 (56)	64 (51)	0.402
Caucasian, *n* (%)	281 (96)	120 (98)	124 (98)	0.549
Transplant indication, *n* (%)				0.106
COPD	120 (41)	51 (42)	53 (42)	
Fibrosis	63 (21)	23 (19)	32 (25)	
Pulmonary hypertension	35 (12)	11 (9)	18 (14)	
Cystic fibrosis	43 (15)	23 (19)	17 (14)	
Other	31 (11)	15 (12)	6 (5)	
Bilateral LTx, *n* (%)	267 (91)	118 (96)	113 (90)	0.057
Time since transplant **, years	8 (4–12)	9 (5–13)	7 (4–11)	0.047
Comorbidities, *n* (%)				
Hypertension	137 (47)	54 (44)	64 (51)	0.276
Dyslipidemia	236 (81)	101 (82)	100 (79)	0.583
Diabetes Mellitus	99 (34)	42 (34)	46 (37)	0.697
Chronic kidney disease	205 (70)	77 (63)	99 (79)	0.006
Obesitas, BMI> 30	33 (11)	12 (10)	14 (11)	0.727
Heart failure	27 (9)	11 (9)	12 (10)	0.874
Immunosuppression, *n* (%)				
Tacrolimus	245 (84)	100 (81)	105(83)	0.372
Cyclosporine	10 (4)	5 (4)	3 (2)	0.455
Azathioprine	57 (22)	27 (22)	22 (18)	0.377
Mycophenolate mofetil	199 (76)	80 (65)	85 (68)	0.686
mTORi	12 (5)	5 (4)	6 (5)	0.781
CLAD	86 (30)	33 (27)	41 (33)	0.324
COVID-19, *n* (%)	146 (50)	49 (40)	54 (43)	0.629
All-cause mortality (%)	20 (7)	3 (2)	17 (14)	0.001
COVID-19 mortality (%)	4 (1)	0 (0)	4 (3)	0.046

Continuous variables are expressed as median (interquartile range). COPD, chronic obstructive pulmonary disease; LTx, lung transplantation; mTORi, mammalian target of rapamycin inhibitors (everolimus or sirolimus). ^#^ Post-COVID-19 Responders 43 (15%) were excluded from analysis: These are patients with a previously negative vaccine antibody response, but a positive antibody response after a positive SARS-CoV-2 antigen test. * *p*-value for the difference between responders and non-responders. ** time between transplant and first vaccination.

**Table 2:** SARS-CoV-2 cumulative antibody response after 1–5 vaccinations.

	Vaccine 1	Vaccine 1–2	Vaccine 1–3	Vaccine 1–4	Vaccine 1–5
Patients with antibody response assessed, *n* (%)	11/292 (4)	249/292 (85)	234/292 (80)	243/292 (83)	220/292 (75)
Patients with pos. antibody response *, *n* (%)	0 (0)	37 (15)	85 (36)	112 (46)	113 (51)
Patients without pos. antibody response ^#^, *n* (%)	8 (73)	197 (79)	117 (50)	88 (36)	58 (26)
Non-response, *n* Low response, *n*	7 1	159 38	84 33	55 33	26 32
Patients with antibody response due to COVID-19, *n* (%)	3 (27)	15 (6)	32 (14)	43 (18)	49 (22)

* Patients who were responders after a given vaccine were considered to be responders to a subsequent vaccine; ^#^ Patients who were non-responders after a given vaccine were considered unknown if their antibodies were not measured after a subsequent vaccine. Vaccine response: IgG level ≥ 300 BAU/Ml. No response: IgG < 300 BAU/mL. Low response: IgG level 50–300 BAU/mL.

**Table 3:** Outcome in SARS-CoV-2 vaccine responders and non-responders with COVID-19.

	Responders with COVID *n* = 49	Non-Responders/Low Responders with COVID *n* = 54	*p*-Value
**Admission, *n* (%)**	4 (8)	9 (17)	0.168
**Death, *n* (%)**	0 (0)	4 (8)	0.046

## Data Availability

The data presented in this study are available on request from the corresponding author.

## Abbreviations

CKD	chronic kidney disease
COPD	Chronic obstructive pulmonary disease
COVID-19	coronavirus disease
ICU	Intensive care unit
ILD	Interstitial lung disease
LTx	Lung transplant/lung transplantation
SARS-CoV-2	Severe acute respiratory syndrome coronavirus-2
